# Palliative Care and Grief Counseling in Peri- and Neonatology: Recommendations From the German PaluTiN Group

**DOI:** 10.3389/fped.2020.00067

**Published:** 2020-02-27

**Authors:** Lars Garten, Marcel Globisch, Kerstin von der Hude, Karin Jäkel, Kathrin Knochel, Tanja Krones, Tatjana Nicin, Franziska Offermann, Monika Schindler, Uwe Schneider, Beatrix Schubert, Thomas Strahleck, The PaluTiN Group

**Affiliations:** ^1^Department of Neonatology, Palliative Neonatology Team, Charité-Universitätsmedizin Berlin, Berlin, Germany; ^2^Department for Content and Development, German Children's Hospice Association, Olpe, Germany; ^3^Association of Premature and At-Risk Born Children, Regional group of Rhineland-Palatinate, Mainz, Germany; ^4^Center for Pediatric Palliative Care, University Children's Hospital, Ludwig Maximilian University of Munich, Munich, Germany; ^5^University Hospital Zürich/Institute of Biomedical Ethics and History of Medicine, University of Zürich, Zurich, Switzerland; ^6^Department of Obstetrics, Klinikum Hanau, Hanau, Germany; ^7^Federal Association of Orphaned Parents and Mourning Siblings in Germany, Leipzig, Germany; ^8^Department of Neonatology and Paediatric Intensive Care, Universitätsklinikum Mannheim, Mannheim, Germany; ^9^Department of Obstetrics, Universitätsklinikum Jena, Jena, Germany; ^10^Roman-Catholic Diocese of Rottenburg-Stuttgart, Department Pastoral Care in Health Care, Universitätsklinikum Tübingen, Tübingen, Germany; ^11^Department of Neonatology and Neonatal Intensive Care, Klinikum Stuttgart, Olgahospital, Stuttgart, Germany

**Keywords:** neonate, pregnancy, life-limiting disease, palliative care, parents

## Introduction

Whenever parents lose their child, it is an enormously emotionally stressful situation for the family, regardless of whether the child is a stillborn or dies later in life. The earlier this painful loss occurs, the more precious becomes every opportunity for the family to spend with their child, providing care as well as saying goodbye (1, 2).

The evidence base in pediatric palliative care is not robust and there is even more paucity of evidence with regard to peri- and neonatology. Yet, palliative care of newborns and grief counseling of families differ significantly from other pediatric palliative care situations with regard to the following aspects (3–9). In case where the diagnosis of a life-limiting disease is established prenatally palliative care and family grief counseling to commence is required before the patient is even born. The pregnant women and the father have to face crucial decisions on behalf of their child and to put into practice their parental responsibility even though they may not yet feel like parents. If the diagnosis of a life-limiting disease is established immediately after birth the complex challenges fall into a particularly sensitive period of parent-child bonding. Specifically, bonding building relationships and becoming a family are required to coincide with the beginning of the mourning process. The situation is characterized by particular psychological burden and often time pressure. Furthermore, prenatal decisions involve health issues of the mother and sometimes the twin. Due to highly dynamics of the clinical situation, neonatal palliative care primarily takes place within the inpatient setting of perinatal centers. In contrast, the focus on pediatric palliative care is at home (10). Due to these factors the newborn child often fails to secure a firm place in its individual family structure and history. For the family and society the child usually remains unreal—as if it had never existed. Since other family members often were unable to get to know the newborn, they do not know the person for whom the parents are grieving. As a result, orphaned parents are at risk of being left alone with their grief and being socially and emotionally isolated. For parents the enduring impact of the life and death of their unborn or newborn child remains to this day scarcely acknowledged up to now.

Albeit these fundamental medical and psychosocial specific issues in perinatal palliative care, up to 2016 professional caregivers in German-speaking countries could neither draw upon evidence nor consensus-based national recommendations for this unique care situation. Our recommendations for palliative care and grief counseling in peri- and neonatology are intended as information and orientation for multidisciplinary care teams and counselors who assist (expectant) parents in these exceptional situations. The purpose is to provide practical guidance and standardization in support and care of newborns in palliative care and their families. The prenatal period, the birth, as well as the time after the death of the child are explicitly included.

The present recommendations do not claim to be able to answer all questions. They rather aim at serving as an orientation, encouraging the international discussion as well as triggering continuous development. They herein refer to situations that may run a similar course, deliberately leaving room for individual adaptation, accommodating the varying needs of affected parents.

We present a short summary of the German recommendations for palliative care and grief counseling in peri- and neonatology and their development process.

## Participants and Methods

Given that palliative care is practiced in a multi-disciplinary approach, these recommendations have been compiled with a participatory approach involving representatives of various professions and specialist disciplines, involved in perinatal care and counseling, as well as bereaved parents. The German expert panel named “The PaluTiN group” (PaluTiN = “Palliativversorgung und Trauerbegleitung in der Peri- und Neonatologie” = Palliative Care and Grief Counseling in Perinatology and Neonatology) was formed in 2016 and came together under the auspices of the German National Association “'Das frühgeborene Kind' e.V.” in order to develop a useful guidebook for contemporary palliative care and grief counseling in the perinatal and neonatal period.

This expert panel was composed of a convenient sample of 12 German speaking members and consisted of 2 neonatologists/palliative care physicians, 1 palliative care physician, 1 palliative care & neonatal intensive care nurse, 1 sociologist, 1 grief counselor in neonatology, 1 clinical ethicist, 1 midwife, 1 specialist in gynecology, obstetrics & perinatology, 1 theologian/chaplain, and 2 parent representatives.

The “PaluTiN group” included experts from a range of different regions of Germany and from Switzerland, with clinical experience in the fields of perinatology, palliative care, bereavement interventions, ethics, and/or pastoral care. They were identified either through their publication and citation record, or through contacts from the professional network of the German National Association “'Das frühgeborene Kind' e.V.”, the German Association for Neonatology and Pediatric Intensive Care, the German Association for Palliative Medicine, or the German Society for Obstetrics and Gynecology.

Respectively, the composition of members of the expert panel was rooted in the central principles of palliative care of working together interdisciplinarily and interprofessionally, integrating the values and life goals of the families concerned. For detailed information regarding all expert panel members and their contribution to different subgroups representing 10 themes of high clinical relevance (see below) please see Supplementary Table 1.

For the development process a modified Delphi-process was used to evaluate the validity of each draft recommendation statement. The Delphi method is a structured communication technique based on bringing together a group of experts in two or more rounds, facilitating the formation of a consensus and recommendations (11).

In total three moderated face-to-face Delphi rounds were held and four additional conference calls were conducted by all 12 members of the expert panel to formulate and agree on each and every recommendation over a course of 18 months. All group members interacted during these face-to-face meetings and in addition by iterative e-mails between each other. All conclusions and recommendations were discussed in detail and step-by step until a full consensus was achieved for each statement. This modified Delphi process with a large proportion of moderated face-to-face communication was chosen, because (i) it builds trust and transparency between the parties communicating, (ii) it enhances productivity by giving attendants the opportunity to brainstorm over a task without the limitations and confines of time, (iii) it allows for immediate very detailed feedback questions and answers between all interprofessional experts and parents, resulting in productive, nuanced discussions and constructive conclusions, and (iv) results in more effective interpersonal fusion of horizons of various experiences of professionals and parents to find common ground (12).

**Figure 1:**
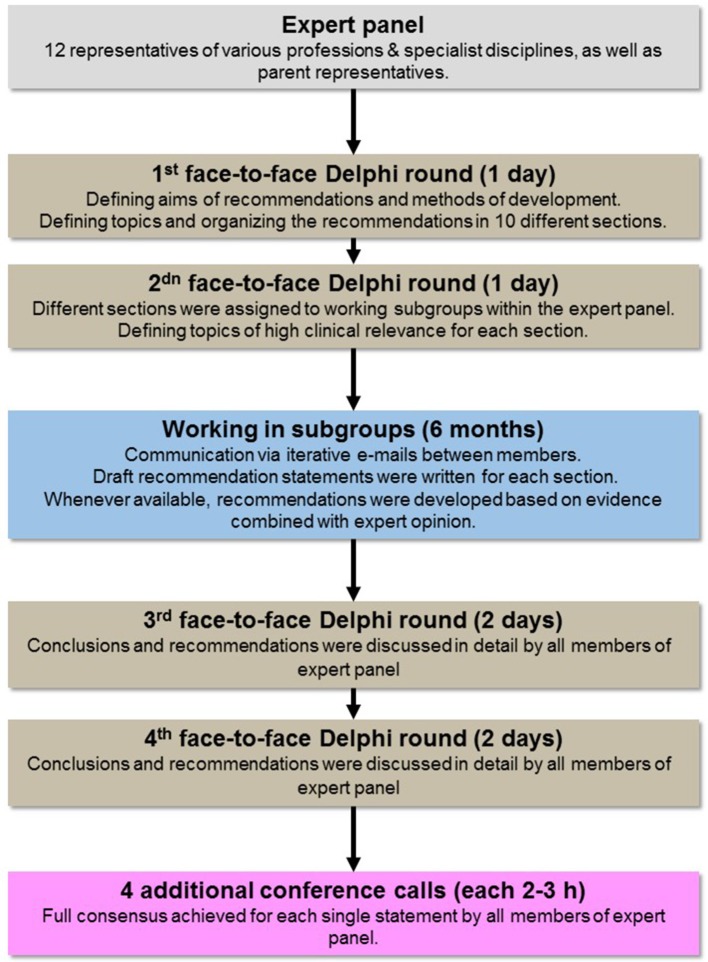
Development process of recommendations.

The development process is summarized in Figure 1.

**Figure 2:**
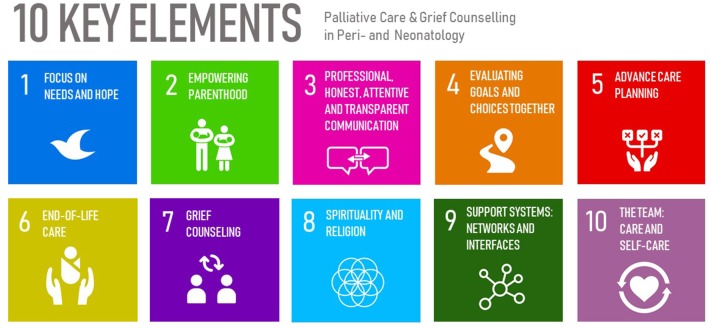
Sections representing topics of high clinical relevance as identified by the expert panel.

The first face-to-face meeting in October 2016 resulted in organizing the recommendations in 10 different sections representing themes of high clinical relevance (Figure 2). We assigned those topics one-by-one to a subgroup of our expert panel with particular experience in that respective sub-area to foster the process of a common discussion and endeavor the draft of a recommendation in that special field. Whenever available, recommendations were developed based on

- guidelines of (inter)national health care and development organizations (13–16),
- systematic reviews, position papers and recommendations (1, 3–6, 6, 17–53)
- individual studies that were identified by reviewing the reference lists of the systematic reviews, position papers and recommendations, and
- textbooks most frequently used in daily German clinical practice (54–62).

The evidence was combined with expert opinion of the panel team members.

## Recommendations

### Focus on Needs and Hope

The diagnosis of a life-limiting illness before or surrounding the birth process plunges unprepared parents and families into a life crisis (53). Yet, parents describe that hope remains and specific requests and desires evolve, such as: getting to know their child and to accompany her on her journey, having time together as a family, ensuring their child does not suffer, or providing a proper resting place for the child after his death (63). Parents sometimes also hope that, contrary to the prognosis, the child's situation will improve beyond what they themselves and the team expect (64, 65). Independent of treatment decisions parents should have the chance to hold on to their hopes until the end, as hope often serves as a source of strength and helps to endure the painful process. Providing information and support can help parents to find their footing and deal with the current situation (33, 48). This requires the readiness of the team to give the parents time and space to recognize their feelings and needs. Parents should be encouraged to express their desires, values, and hopes. The individuality of each child and each family has to be respected. Their desires and needs should be addressed in a respectful, open and active manner. The family should be supported and encouraged to maintain a regular everyday routine as far as possible despite the extreme situation. Support in organizational matters (e.g., care of siblings, letter of excuse, domestic help) can provide families additional latitude.

### Empowering Parenthood

When birth and death coincide, parents have little time to grow into their role as parents, get to know their child and give it a place within their family. Therefore, the child's care team should support, actively promote and protect the parent-child relationship (6).

Parents who fear the bonding process to their dying newborn may be helped to realize that evading the pain of bereavement often only appears to make parting easier. Parents should be encouraged to commit emotionally to the child in order to allow them to take their first steps in dealing with the loss through a consciously designed parting. They need support and skills of staff in dealing with pain and avoidance reactions. Even if a prenatal diagnosis of a life-limiting disease has been made, it is important to create space for the bonding process (66). Indeed, the pregnancy can become a significant family life event and an important time to remember.

Parents should be supported in getting to know their child and giving her a place within their family. A newborn baby has not yet a place in the family as a living infant (67), and family members and others may not have formed strong attachments when the child is born. Generally fetal or neonatal loss is socially less recognized and parents feel lonely and abandoned (68, 69).

All parental instincts and efforts to care for their child should be valued and actively encouraged (70). In this limited time, parents need as many opportunities as possible to be with their child, get to know him and grow into their parental role. Parents need to be encouraged to involve siblings and other important people from their family and social environment and to offer them the opportunity to get to know the child, too. The staff's natural and caring approach to the dying or deceased child can serve as a role model for parents. It can make it easier for them to approach their dying or deceased child and deepen their bond. By providing the child a place within the family and circle of friends, the family members come to know for whom it is that the parents are grieving. They are thus better able to support the mourning family with understanding and long-term support. For the grieving parents, this reduces the risk of social and emotional isolation, as well as the great worry that their child will be forgotten.

### Communication—Professional, Honest, Attentive, and Transparent

The relationship between family and care team is rooted in partnership and professionalism. The communication should be professional, ongoing, honest, empathetic, consistent, and transparent (44, 68, 71, 72). Parents should be confident that they are being listened to carefully and that no information is being withheld from them. The fundamental demeanor of the care team members is characterized by acceptance, respect, esteem, empathy, attentiveness and authenticity (69).

Crisis talks and other encounters need a protected, appropriate setting (65, 73). They need to take place in a calm environment, if possible free of time pressure and disturbances. Particularly important conversations should be scheduled as dependably as possible in order to give both parents the opportunity to participate and to prepare themselves. In critical or changing situations, conversations should be timely, not delayed and contact persons available at any time. At the same time, the possible desire of parents not to know should be respected. In addition, parents should be able to trust that they can tell and ask all members of the health care team anything without having to fear negative consequences for themselves and their child. It is important that they are accepted in their attitude and that their ambivalence, which often occurs in these situations, be respected.

Professionals may at times be especially challenged by emotionally stressful discussions and react with a degree of uncertainty, hesitancy or even avoidance. That is why they require both individual and team skills, special abilities and attitudes, as well as continuous reflection, training and possibilities for debriefing and supervision (46).

### Evaluating Therapeutic Goals and Treatment Choices Together: Stepwise, Sound, and Sustainable

A continuous, open and honest exchange of information, values, goals and treatment options between the expectant mother or the parents and the care team is the foundation for sustainable decisions (74). The active process of shared decision-making should enable the parents and the specialists involved to understand the situation in all of its dimensions (75, 76). Parents should be supported in making clear long-term decisions that are in the best interest of their child (77–79).

The diagnosis or the symptoms of a severe life-limiting illness entail complex challenges for treatment decisions and interventions (80). Parents have the right and, in the majority of cases, the desire to take an active part in the decisions concerning their child (70, 81). They are continually encouraged to determine for themselves the degree of their involvement. For the care team this means sustainably and constantly sharing ideas with the parents, listening to them, getting to know their values and, if desired by the parents, shaping the decision-making process together with them as equal partners in the process (shared decision-making) (23, 78). In decisions made during pregnancy, the mother's right to self-determination is the legal priority. At the time of birth, in Germany, parents are entitled to define the best interest and well-being of the child also with regard to health care within broad legal limits as a natural legal custodial right. It is therefore their task to decide which steps are to be taken regarding treatment decisions based on their child's best interest.

The basic prerequisites for the decision-making process are open, understandable, empathetic communication and a comprehensive exchange of information with the parents (32, 82). The support provided by the care team consists of a careful, stepwise examination of all options together with the pregnant mother/parents (30, 83). Medical information should be communicated in a clear and comprehensible manner. Local, national, and international studies on prognosis and treatment options should be taken into account as far as possible (34), for example in the form of evidence-based decision aids.

It is essential to include, in addition to the physician's perspective, the expertise of midwives, nurses, therapists (physiotherapists, music therapists, etc.) and specialists in psychosocial and spiritual care (45, 84). This offers the opportunity for a holistic perspective including the expertise of all participants, making it easier for parents to assess the impacts of their decision on the future life of their child and family.

Once medically, ethically, and legally sound parental decisions have been made, they should be respected by all concerned, even if the care team would prefer a different treatment option. In very rare cases, if a viable common decision cannot be reached, the ethical principle of causing no harm can become the guiding principle for treatment.

It is important and helpful to formulate a transparent therapeutic plan for each step and for all participants. Formal documentation of the process and decisions facilitates the interdisciplinary exchange of information and can prevent loss of information. It should always be considered that parents may re-evaluate previous decisions during the subsequent course of the treatment (e.g., after the birth of their child) (85). The care team may also need to re-examine changes as treatment progresses (86). Therefore, open discussions within the team and with the parents, ideally with the same contact persons, are always necessary.

### Advance Care Planning

Advance care planning, first developed as a new concept in the late 1990s (87, 88) for planning ahead for situations of health crisis and incapability of decision-making with patients being fully capable of decision-making with family and caregivers (16, 88, 88, 89). It is a concept which can be adapted for discussing and planning future treatment decisions and interventions during the peri- and postnatal situation of a child with a life-limiting illness (90, 91). Medical and non-medical issues are thoroughly evaluated and discussed regarding possible disease trajectories before and after birth (63). This process also includes summarizing decisions in writing in a transparent and easily understandable manner and to ensure its implementation. Therefore, the advance care plan needs to be communicated to all persons involved in taking care for the pregnant women and the child.

Outside of curability, situations will inevitably arise during the clinical course in which alternative therapeutic goals and treatment options may suggest individual courses of action (92). Supplementing the current treatment plan, advance care planning is utilized together with the parents to discuss and define the measures to be taken in the event of future developments thoroughly and without time pressure (93). It encompasses the discussion of medical and non-medical issues that already play a role or may become important in the course of treatment e.g., the integration of and the care for the siblings (94–97). These physical, social, psychological, and spiritual needs of the child and its family should be carefully identified in the discussion and considered by the multi-professional team. Parents should be encouraged to formulate their wishes and goals (98).

Basically, an advance care plan addresses the following stages in the course of the illness (16): (1) disease progression, i.e., when the patient's condition slowly deteriorates, (2) acute life-threatening crises, (3) altered baseline status following an acute crisis.

Outside of supporting parents by preparing emergency and crisis situations, it is the goal to identify central wishes and concerns and pave the way for shared decisions for future interventions. It should be sought to document the summary of the advance care discussion in writing. The documentation should include specific details of the discussion process, the therapeutic goals and measures.

Especially for an acute emergency situation, it is important to specify in detail whether life-sustaining measures should be initiated, and if so, which ones and to what extent. It is also helpful to include a “neonatal emergency care directive,” analogous to the US American “Physician order for life sustaining treatment (POLST”) form in the written document (43). This should be signed by the physicians and, if possible, by the parents in order to provide the first responders and specialists involved with important information and clear instructions (99).

For palliative care at home it is particularly important that the treatment plan anticipates measures for symptom control for foreseeable crises. For example, medication required under certain circumstances should be provided and its administration ensured.

Effective interfaces among interdisciplinary care team members and other disciplines are a prerequisite to ensure the sharing of information and implementation of the treatment plan (18). The cooperation of all care providers including the broader family, if whished by the parents, aims at avoiding loss of information and a solid base for action also during crisis. This is considered as being essential for confidence and trust of families (100).

Notwithstanding any ambivalence among parents and specialists (101), this stepwise process allows to discuss jointly and repeatedly with the parents and may offer benefits for professionals and parents. Professionals in pediatric palliative care stressed the better sense of security, the improvement of the quality of care and the respect of individual wishes (102). Parents described positive effects, particularly to ease the feeling of loss of control, to appreciate the step-by-step approach and to feel better prepared by empowerment (92, 103).

### Comfort Care Towards the End of Life

The essence of palliative care for newborns with life-limiting illnesses is not upon life prolongation at any cost, but rather upon providing the best quality of life, emphasizing the well-being of the child and her family (20). In any medical decision, the best interest of the child has the highest priority (41, 104). This means that no treatment may be carried out that burdens the child without providing him with a tangible benefit. All diagnostic and therapeutic procedures should be critically examined and reviewed in this respect (22). This also includes life-supporting measures such as respiratory assistance, supplemental oxygen, infusion therapy, partial or complete parenteral feeding or feeding via nasal gastric/duodenal tubes (49, 105).

The dying child should be carefully and systematically assessed for any evidence of pain and other signs of suffering such as shortness of breath, restlessness and anxiety. The parents' assessment of the child's condition should be taken into account. Appropriate pain management and symptom control (106) through non-pharmaceutical (107–109) and pharmaceutical measures (27, 31) should be available at any time during the dying process and at any location [in the delivery room (29), in the neonatal intensive care unit, in the children's hospice, or at home]. Symptom-relieving medication can be administered via existing superficial, central or indwelling intravenous catheters, as well as orally, nasally, rectally, or transdermally. Intramuscular injections as well as single shot venous or subcutaneous injections should be avoided.

The administration of drugs such as opiates and benzodiazepines with the primary aim of alleviating suffering and symptoms should proceed despite possible respiratory or circulatory depressing side effects (37). Here it is essential to maintain full transparency with the parents regarding the medical treatment. The immediate care of the child is exclusively based upon its individual needs (“optimal care”). The focus should be to support the child's well-being, prevent symptoms or alleviate them, e.g., closeness and skin contact, hearing familiar voices or melodies, positioning to ease breathing, or avoiding hunger and thirst.

It should be noted that parents are the most important caregivers for the child. They should be supported in exercising their parental autonomy and caring (6). With their experience, the care team should provide the parents with reassurance and calmness while escorting their child through the final period of life. Even during this highly emotional time, the creation of lasting memories can be important. This should be supported and offered individually, attentively and sensitively, without disturbing family intimacy.

Human closeness remains the highest priority while the newborn is dying. In virtually every case, the supportive presence of the parents should be made possible. If the parents cannot be reached or for personal reasons are not able to be present, the necessary closeness and human attention should be provided by another family member, friend or someone from the team. Especially during dying, every family's right to privacy should be taken into account. The parents can decide whether to remain alone with their child or rather prefer that a team member be present for support. Family members requested by the parents should be given expeditious access to the child, if not already present. This applies especially to siblings.

Once the final stages of the dying process have begun, it is the team's primary task to stay in attendance without being intrusive. The goal is to enable the child to die in comfort and with dignity. Parents should be braced for understanding and anticipating possible symptoms, reflexes and behavior patterns of the dying child in order to avoid confusion and fear. Individual variability in the duration of the dying process should also be pointed out.

### Grief Counseling

Grief is the normal reaction to a significant loss. For affected parents, grief at the beginning of life often means having to say goodbye to their child before they have welcomed him into the world and into their family (6). The parents' mourning process often begins with the diagnosis (69, 110), and requires a personalized, reliable and on-going support system from the onset (2). Supportive grief counseling fosters the parent-child relationship as well as the bonding process within the entire family (25). It also offers help toward self-help, for practical organizational needs, for the development of necessary support systems and the transition back into the home environment (19). Grief counseling carefully seeks out paths and taps resources for a healthy continuation of an emotionally, psychologically, socially, spiritually and physically stable life. The long-term goal of grief counseling is to enable grieving families to integrate the loss of the child into their lives and family history (25).

At the beginning of their bereavement journey, parents need a proactive, accessible contact. At all times, they are free to accept or reject individual offers of support. Offers of grief counseling should be periodically repeated, as the parents' mental state, needs and situation may often change with time. Grieving parents and their families need reliable and readily available contact persons (100). These should encourage them to express their thoughts, fears and challenges and to ask questions at any time. The time spent with the child is so brief, that every opportunity to create and deepen attachment counts. Every experience during the life and the death of the child is invaluable, since these memories serve to preserve the entirety of a family's history.

Fears of deepening attachment to the dying or deceased child can be counterbalanced by positive narratives from other parents in the same situation. Hearing that grief is generally not worsened by repeated encounters can encourage parents to make use of the time while their child can still be experienced. However, parents maintain the choice at any time to not see their child.

Personnel, staffing, environmental, and organizational practices should be designed to enable families to spend time with their child up until the burial. This also includes the opportunity to invite siblings and important family members to get to know the sick, dying or deceased child and thereby better understand whom it is that the parents grieve.

From the moment of diagnosis, parents live through an exceptional situation. As a result, it may be necessary during grief counseling to raise topics that the parents themselves will seldom initiate. This includes aspects such as: inclusion of siblings and addressing their grief, individual differences in expressing grief and coping with the potential for conflict in the relationship (111), ambivalent feelings in cases where not all the babies from a multiple birth survive, dealing with feelings of insecurity and social isolation, preparatory steps for reentry into daily life, midwife support and services, finances (e.g., funeral costs, loss of income).

In order to ensure that outpatient grief counseling may continue, it is necessary to identify potential resources (e.g., regional grief counseling, psychosocial services, midwives, outpatient children's hospice services, community-based, and church services) with the parents, allowing them access to a support network (6, 26). In situations where the grief is intensifying, the mourner should be offered a referral for appropriate and specialized assistance.

### Spirituality, Religion, and Pastoral Guidance

When birth and death occur so closely together, it creates an existential crisis. Life plans are destroyed. Assumptions about life, its meaning, its values and its spiritual-religious grounding are suddenly called into question (52). It is the task of the team to continually offer spiritual and religious support to the involved families. This may include a variety of very different types of support, and applies to all participants, regardless of their spiritual or religious orientation.

People enquire into the mystery of life and seek its meaning. This is a characteristic trait of human existence, which we call spirituality. Pastoral care is attentive to the spiritual and religious needs of parents. Many in these situations hold onto their faith as a source of strength, its encouragement through pastoral care offering an additional resource for coping. If one's own faith is not (or is no longer) perceived as supportive and comforting, however, parents may consider pastoral care as an additional burden. The question of guilt, even if it is not expressed outright, can nevertheless be an important issue, and one to which the care team and especially the pastoral care giver should be attuned. In their approach and manner the pastoral care workers convey to their clients that they stand by them and they are searching for answers together. They are sensitive to the spiritual and religious needs of their clients, and do not offer ready-made answers. Their spirituality is recognizable and truthful; they wish never to promote their own ideas, much less to impose them.

It is necessary to distinguish between the broader spiritual-pastoral and the narrower religious-pastoral services. Baptism, blessing, prayer, as well as greeting and farewell rituals place the child in his uniqueness at the center of the parents and care team, regardless of his state of health. At the same time, they remind us of the lack of control over life despite all the technological possibilities. Rituals are an expression of love and appreciation. They open up another dimension of familial experience in the midst of all the medical treatments. They demonstrate appreciation for the child, connect him to the community and thereby give him a permanent place there, even if he dies (24). Rituals can connect the parents and the team with each other.

Pastoral workers can additionally play a helpful role for the care team in coping with their own work stress, and with their own spiritual and religious questions and needs. In these cases, talking, as well as rituals may provide relief and support.

### Support Systems: Networks and Interfaces

Palliative care of newborns, as well as counseling and grief support for affected parents begins with the diagnosis, which is sometimes already established prenatally. For this reason, an interdisciplinary, cross-sectoral network is needed from the outset, consisting of outpatient and inpatient care providers (112). Families and network partners require that responsibilities be clear, transparent and well-defined in order to ensure optimum care (100). This also includes the designation of a coordinator whose responsibilities include being aware of possible gaps in care or communication and who can propose solutions (38). There is no universal standard for the structure of a well-functioning network of support systems. Regional solutions are therefore required based upon available facilities and human resources. The evaluation of networking performance should be defined individually as well as structurally, and should fundamentally be inclusive of all parties.

### The Team Duality: Care and Self-Care

Perinatal-palliative care is particularly challenging both personally and existentially. It therefore requires appreciative respect and special support. Optimal support for the patients and relatives will only be possible if the resources of the team members are effectively strengthened and their resilience is fostered (47). In order to take good care of the patients and families entrusted to them it is necessary to recognize, in accordance with the updated Declaration of Geneva in 2017 (113), one's own needs and to take them seriously. Only those who take good care of themselves can continue to take good care of others.

High quality palliative care and grief counseling also require professional objectivity in order to assess the needs of the child and family and to be able to take appropriate action. Palliative support personnel should establish sufficient closeness to allow trust, but nevertheless maintain adequate perspective and a sense of boundary in order to recognize their own limitations.

The team members should be involved in the development and planning of therapeutic options. A shared understanding of the different points of view lessens the moral distress. This equally suggests that every team member has the right to initiate a discussion about therapeutic goals.

Regularly held discussion forums within the institution help give every team member the opportunity to address events that have occurred in a constructive, respectful and empathetic manner. Within the team, attention should be given to monitor if each member remains able to cope with the strain of caring for dying and dead children, as well as for their families. Whenever this tolerance is exceeded, the responsibility should be assumed by another team member.

Rituals and developing a culture of farewell, professional and personal interaction, as well as continued education and supervision contribute to professional self-care (114). Each team member has the obligation not only to take care of himself, but also to look out for other team members. However, the institution and the facility have an obligation to provide the personnel, space and organizational resources necessary to allow these requirements to be implemented.

## Discussion

Peri- and neonatal palliative care and supportive grief counseling is complex and differs in some ethical, medical, and psychosocial aspects from pediatric palliative care. Similar to pediatric palliative care it requires a specialized multidisciplinary approach to meet physical, psychosocial, and spiritual needs of a child and his family. In the last decade, standards, guidelines and quality improvement strategies have been developed for pediatric palliative care. This has been a major contribution to the integration of palliative care into daily practice and has improved its quality. In contrast, palliative care and grief counseling in peri- and neonatology have made a much slower progress. Local initiatives have tried to give guidance in neonatal palliative care, but standardization of care is lacking. By using an expert panel involving health care professionals of different backgrounds and bereaved parents, we provided a broad participatory approach on a national level. The recommendations presented here are based on the combination of the evidence available and a national expert consensus.

Despite the fact, that the evidence level of most of our recommendations is low due to the lack of rigorous evidence, these first results have an important role in guiding health care providers to the optimal clinical care of affected children and families.

However, we do also acknowledge the limitation of the methods of evidence-based medicine in our field, where values of individual families, a central “goals of care”-approach and ethical reasoning are paramount to decision-making and practice. Although within a society as well as between societies value systems differ, with no existing “objective” common agreement on e.g., what quality of life is or dignity, respectively, person-centered care and trust to define the best interest of the severely ill child primarily by families, based on deep respect for parental autonomy, suffering and life goals as the basis for decision making, are widely shared around the world. The description of core concepts of excellent value-based medicine including palliative care, entailed in the current.

German recommendations add specific details of the PaluTiN expert consensus to the international debate aiming to improve the care for children diagnosed in the pre- or postnatal period with life-threatening conditions and for their families.

Finally and self-evident these recommendations do not claim to be able to answer all questions. They should rather serve as an orientation, encouraging discussion, and continuous development. Further recommendation updates are required to improve the quality of palliative care and grief counseling in peri- and neonatology.

Full text version (original German version and translated English version) of recommendations is available on: https://www.fruehgeborene.de/fuer-fachleute/palliativversorgung-und-trauerbegleitung.

## Author Contributions

LG, MG, KH, KJ, KK, TK, TN, FO, MS, US, BS, and TS contributed to the working group and/or to the discussions of the findings of the working group and all offered amendments to the previous drafts and/or agreed the manuscript.

### Conflict of Interest

The authors declare that the research was conducted in the absence of any commercial or financial relationships that could be construed as a potential conflict of interest.

## Supplementary Material

Supplementary Table 1 Detailed information on all members of the PaluTiN group.

## Abbreviations

NICU: neonatal intensive care unit.
