# Supplementary material for: Palliative Care and Grief Counseling in Peri- and Neonatology: Recommendations From the German PaluTiN Group
Source: Front Pediatr. 2020 Feb 27;8:67. doi: 10.3389/fped.2020.00067 (PMC7058113; doi:10.3389/fped.2020.00067)
Supplement: Supplementary Table 1 — Detailed information on all members of the PaluTiN group. [file Data_Sheet_1.PDF]

Name	Profession/workplace	Member of association/society	Member of subgroup No.
Dr. med. Lars Garten	Senior Physician at the Clinic for Neonatology and Head of the Palliative Neonatology Team at the Charité University Hospital, Berlin	- German Association for Neonatology and Pediatric Intensive Care (GNPI)<br>- German Association for Palliative Medicine (DGP)<br>- German Society of Pediatrics and Adolescent Medicine (DGKJ)	*4, 5, 6, 9*
Marcel Globisch, M.A.	Sociologist, Department Head for Content and Development at the German Children's Hospice Association, Head of the Division for Hospice Work and Palliative Care for Children of the German Hospice and Palliative Care Association	- German Hospice and Palliative Care Association	*8, 9, 10*
Kerstin von der Hude	Psychosocial parent counselor in neonatology and member of the neonatology palliative team at the Charité University Hospital, Berlin. Systemic couple and family counselor (DGSF), ethics counsellor in health care (AEM), grief counsellor.	- German Association for Neonatology and Pediatric Intensive Care (GNPI)	*2, 7*
Karin Jäkel	Orphaned mother of an extremely premature twin child, senior lecturer for protestant religious education, founding member and board member of the regional association „Früh- und Risikogeborene Kinder Rheinland-Pfalz" e.V. (Premature and At-Risk Born Children Rhineland-Palatinate)	- Association for Premature and At-Risk Born Children Rhineland-Palatinate	*1, 2, 8,*
Dr. med. Kathrin Knochel	Palliative physician at the Children's Palliative Center Munich, Dr. von Haunersches Children's Hospital, University Hospital of Munich	- German Association for Palliative Medicine (DGP)<br>- German Interdisciplinary Association of Critical Care and Emergency Medicine (DIVI)	*1, 4, 5, 6, 9*
Prof. Dr. med. Dipl. Soz. Tanja Krones	Head Physician for Clinical Ethics at the University of Zurich Hospital/University of Zurich, President of the International Society for Advance Care Planning, Member of the Board of the Central Ethics Commission of the German Medical Association	- International Society for Advance Care Planning (ACP-i)<br>- Board of the Central Ethics Commission of the German Medical Association (ZEKO)	*4, 5*
Tatjana Nicin	Midwife, Representative of the German Midwifery Association, Dipl. Educator for Special Needs, IBCLC Breastfeeding and Lactation Consultant, Nursing Department of Obstetrics in the Hanau Clinic	- German Midwifery Association (DHV)	*3, 6*
Dr. Franziska Offermann	1st Chairwoman of the Federal Association of Orphaned Parents and Mourning Siblings in Germany (VEID), grief counsellor (BVT), trauma consultant (DeGPT/BAG-TP), coach	- Federal Association of Orphaned Parents and Mourning Siblings in Germany (VEID)<br>- Federal Association of Grief counselling (BVT)	*2, 3, 7, 10*

Monika Schindler	Specialist pediatric nurse, M.Sc. (Palliative Care), nursing director neonatology and pediatric intensive care unit, University Hospital of Mannheim	- German Association for Neonatology and Pediatric Intensive Care (GNPI)	*6, 7, 10*
Prof. Dr. med. Uwe Schneider	Specialist in gynecology and obstetrics, perinatal physician, Professor of Prenatal Diagnostics and Fetal Physiology and Senior Physician of the Clinic for Obstetrics at the University Hospital of Jena	- German Society for Obstetrics and Gynecology (DGGG)	*3, 4, 6*
Beatrix Schubert	Theologian (catholic theology), since 2004 pastoral assistant in the clinic pastoral care unit at the University Hospital of Tübingen, responsible for the Women's Hospital and the Department of Neonatology of the Children' s Hospital		*8*
Dr. med. Thomas Strahleck	Neonatologist, palliative physician, clinical ethics consultant, crisis resources management instructor, senior physician in Pediatrics 4 at the Stuttgart Olga Hospital	- German Association for Neonatology and Pediatric Intensive Care (GNPI)<br>- German Association for Palliative Medicine (DGP)<br>- German Society of Pediatrics and Adolescent Medicine (DGKJ)<br>- Academy of Ethics in Medicine (of the German Medical Ethics Society) (AEM)<br>- German Interprofessional Association of Advance Care Planning (DIV-BVP)<br>- German Interdisciplinary Association of Critical Care and Emergency Medicine (DIVI)	*1, 4, 5,*

Legend.
Subgroups: 1. Focus on needs and hope, 2. Empowering parenthood, 3. Professional, honest, attentive and transparent communication, 4. Evaluating goals and choices together, 5. Advance care planning, 6. End-of-life care, 7. Grief counselling, 8. Spirituality and religion, 9. Support systems: networks and interfaces, and 10. The team: care and self-care.
